# TGF‐β/Smads signaling pathway, Hippo‐YAP/TAZ signaling pathway, and VEGF: Their mechanisms and roles in vascular remodeling related diseases

**DOI:** 10.1002/iid3.1060

**Published:** 2023-11-07

**Authors:** Hui Liu, Mingyue Sun, Nan Wu, Bin Liu, Qingxin Liu, Xueli Fan

**Affiliations:** ^1^ Department of Neurology Binzhou Medical University Hospital Binzhou China; ^2^ Institute for Metabolic & Neuropsychiatric Disorders Binzhou Medical University Hospital Binzhou China

**Keywords:** Hippo‐YAP/TAZ, TGF‐β/Smad, TGF‐β‐1, vascular remodeling, VEGF

## Abstract

Vascular remodeling is a basic pathological process in various diseases characterized by abnormal changes in the morphology, structure, and function of vascular cells, such as migration, proliferation, hypertrophy, and apoptosis. Various growth factors and pathways are involved in the process of vascular remodeling. The transforming growth factor‐β (TGF‐β) signaling pathway, which is mainly mediated by TGF‐β1, is an important factor in vascular wall enhancement during vascular development and regulates the vascular response to injury by promoting the accumulation of intimal tissue. Vascular endothelial growth factor (VEGF) has an important effect on initiating the formation of blood vessels. The Hippo‐YAP/TAZ signaling pathway also plays an important role in angiogenesis. In addition, studies have shown that there is a certain interaction between the TGF‐β/Smads signaling pathway, Hippo‐YAP/TAZ signaling pathway, and VEGF. Many studies have shown that in the development of atherosclerosis, hypertension, aneurysm, vertebrobasilar dolichoectasia, pulmonary hypertension, restenosis after percutaneous transluminal angioplasty, and other diseases, various inflammatory reactions lead to changes in vascular structure and vascular microenvironment, which leads to vascular remodeling. The occurrence of vascular remodeling changes the morphology of blood vessels and thus changes the hemodynamics, which is the cause of further development of the disease process. Vascular remodeling can cause vascular smooth muscle cell dysfunction and vascular homeostasis regulation. This review aims to explore the mechanisms of the TGF‐β/Smads signaling pathway, Hippo‐YAP/TAZ signaling pathway, and vascular endothelial growth factor in vascular remodeling and related diseases. This paper is expected to provide new ideas for research on the occurrence and development of related diseases and provide a new direction for research on the treatment of related diseases.

## Graphical abstract

TGF‐β/Smads signaling pathway, Hippo‐YAP/TAZ signaling pathway, and vascular endothelial growth factor: their mechanisms and roles in vascular remodeling related diseases.

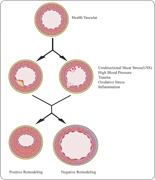

## 1 TRANSFORMING GROWTH FACTOR‐β/SMADs SIGNALING PATHWAY

### 1.1 Transforming growth factor‐β/Smads signaling pathway function

The transforming growth factor‐β (TGF‐β)/Smads signaling pathway plays an indispensable role in the growth and development of embryos, the maintenance of tissue homeostasis, and the proliferation, differentiation, and migration of cells. The pathway was originally studied in the fields of inflammation, tissue repair, and embryonic development. In recent years, TGF‐β has been shown to play an important role in the regulation of cell growth, differentiation, and immune function.^1^

### 1.2 TGF‐β/Smads signaling pathway elements

#### 1.2.1 Ligands and receptors of the TGF‐β/Smads signaling pathway

The TGF‐β superfamily has more than 40 members, including TGF‐βs, bone morphogenetic protein (BMP), activin, and related proteins.^2^ These members have a common dimeric structure.^3^ TGF‐β is secreted in both paracrine and autocrine ways and binds to receptors to regulate the activation or inhibition of its pathways.^4^ TGF‐β receptors have three types: type I, type II, and type III receptors. TGF‐β acts by binding to TGF‐β type II receptors (TβR II) and type I receptors (TβR I) and then activates downstream signaling.^5^, ^6^ However, the Type III receptor inhibits the TGF‐β signaling pathway by isolating TGF‐β.^7^

#### 1.2.2 Members and roles of downstream molecules in the TGF‐β/Smads signaling pathway (Smad family)

Smad transcription factors are the core of the TGF‐β signaling pathway. The TGF family binds to TβRI and TβRII and forms a complex. To date, a total of eight different Smad proteins have been identified in mammals and are divided into three subfamilies according to their functions: receptor‐regulated Smads (R‐Smads), universal chaperone Smads (Co‐Smads), and inhibitory Smads (I‐Smads). Under general conditions, R‐Smads are mainly located in the cytoplasm, I‐Smads are mostly located in the nucleus, and Co‐Smads are distributed in the nucleus and cytoplasm.^8^ The R‐Smad family includes Smad1, Smad2, Smad3, Smad5, and Smad8. Smad4 belongs to the Co‐Smad family.^9^ Smad6 and Smad7 are members of the I‐Smad family, which plays a key role in inhibiting TGF‐β‐mediated signaling. I‐Smads can antagonize TGF‐β/Smads signaling by inhibiting the activation of R‐Smads.^10^

### 1.3 Regulatory mechanisms of the TGF‐β/Smads signaling pathway

**Figure 1:**
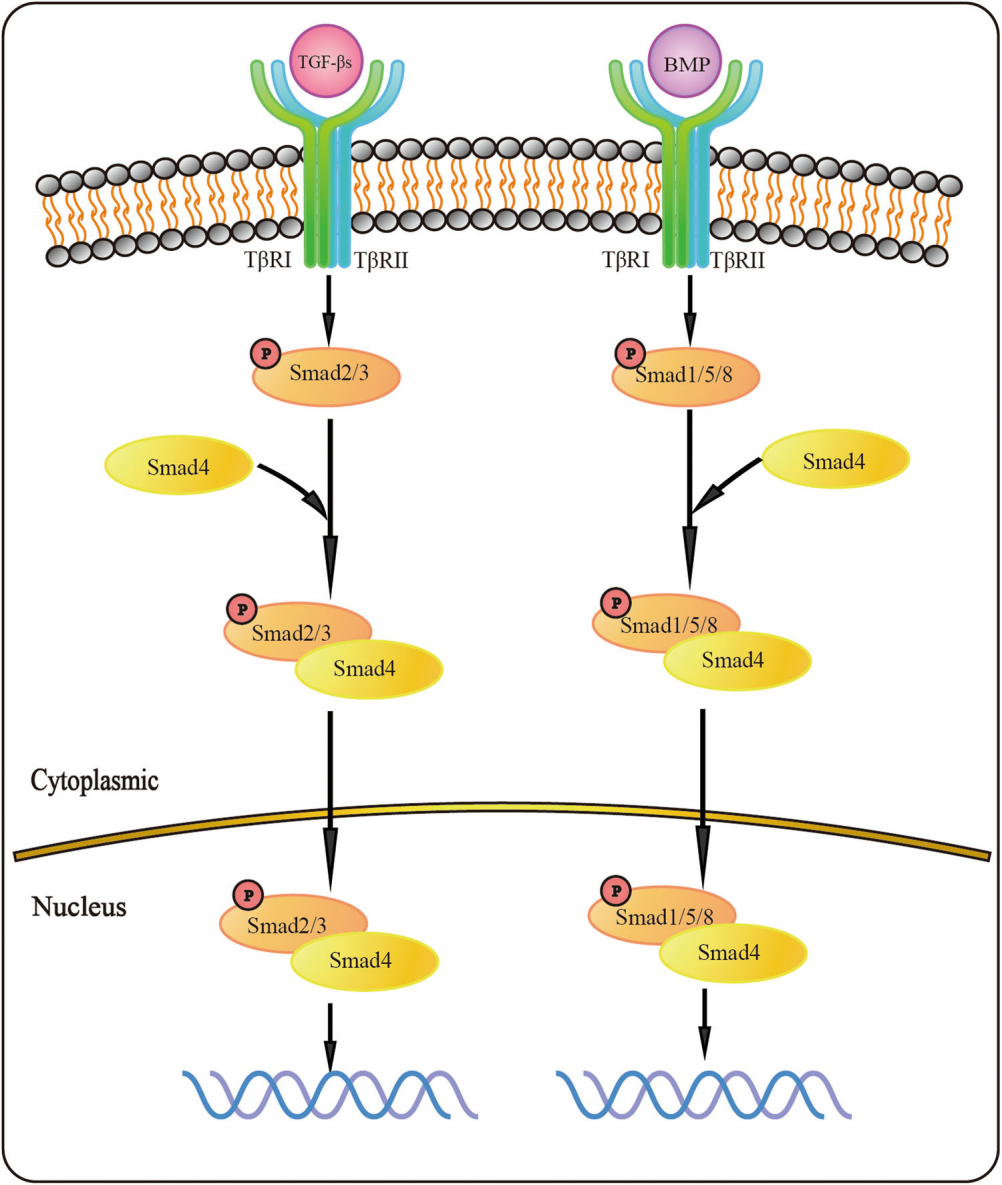
The classical TGF‐β signaling pathway. In the classical TGF‐β pathway, TGF‐β family ligands and receptors bind at the plasma membrane level and then activate and phosphorylate the corresponding R‐Smads protein downstream, namely Smad2/3 or Smad1/5/8. Phosphorylated R‐Smads combine with Co‐Smad (Smad4) to form a complex, which is transferred into the nucleus and mediates gene transcription and expression. TGF‐β, transforming growth factor‐β.

TGF‐β1, TGF‐β3, and activin have high affinity for the type II receptor. Therefore, a unique interface can be generated by first forming a complex with TβR II and then recruiting TβR I to the complex. In contrast, TGF‐β2 has a very low affinity for TβR II. Therefore, TβR I and TβR II are required to first form a complex or coreceptor, such as betaglycan, which facilitates TGF‐β2 assembly within the complex.^11^ The TGF‐β signaling pathway can be activated through the classical Smad pathway and the non‐Smad pathway.^4^ The main focus of this review is on the role of the Smad‐mediated classical TGF‐β/Smads signaling pathway in vascular remodeling. In the classical Smad pathway, complexes formed by the binding of receptors and ligands at the plasma membrane activate the phosphorylation‐dependent signaling of downstream mediators, mainly Smad proteins, and mediate oligomer‐dependent signaling of ubiquitin ligases and intracellular protein kinases under the action of receptor kinases. In the classical Smad signaling pathway, the downstream pathways activated by different ligands are also different (Figure 1). For example, TGF‐βs and activin usually induce the phosphorylation of Smad2 and Smad3, while BMPs usually induce the phosphorylation of Smad1, Smad5, and Smad8.^11^ Phosphorylated Smads (R‐Smads) bind with Smad4 to form heteromeric complexes and transfer them to the nucleus, where Smads and other signaling proteins mediate regulatory signals that control target gene expression, multilevel RNA processing, mRNA translation, and nuclear or cytoplasmic protein regulation.^12^, ^13^

**Figure 2:**
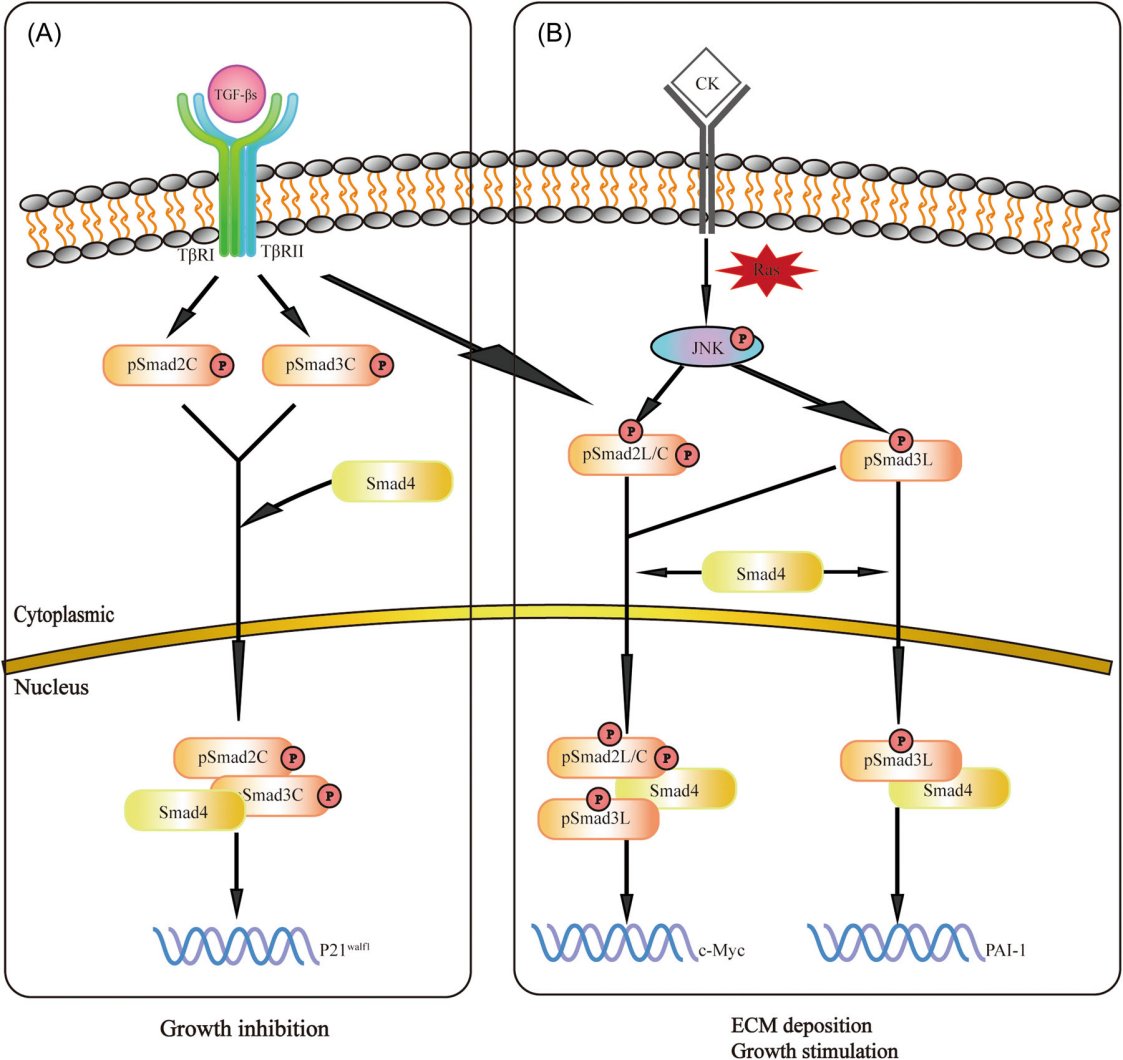
Different smad2/3 phosphorylation sites. (A) TβRI phosphorylates COOH‐tail serine residues of Smad2 and Smad3. Both pSmad2C and pSmad3C translocate with Smad4 to the nucleus. Smad2/3/4 complex binds the p21^waf1^ promoter and suppresses cell growth. (B) Pro‐inflammatory cytokines (CKs) such as tumor necrosis factor‐α activate c‐Jun N‐terminal kinase (JNK), which phosphorylated the linker regions of Smad2 and Smad3. PSmad3L translocates with Smad4 to nucleus and binds plasminogen activator inhibitor type 1(PAI‐1) promoter. PSmad2L is localized in the cytoplasm, and Smad2 translocates to the nucleus only after COOH‐tail phosphorylated by TβRI. PSmad2L/C in cooperation with pSmad3L and Smad4 stimulated PAI‐1 transcription and ECM deposition. In addition, pSmad3L upregulates c‐Myc and stimulates cell growth.^14^

In the Smads‐mediated signaling pathway, phosphorylated Smad2 and Smad3 also have positive and negative regulating effects (Figure 2). Both Smad2 and Smad3 have a conserved MH1 domain that binds DNA, and a conserved MH2 domain that binds receptors, a partner Smad4 and transcriptional co‐activators.^13^ The two domains are separated by more divergent linker regions. The linker regions of Smad2/3 contain serine/threonine residues, and each site is phosphorylated by a specific kinase. For example, TGF‐β is more readily phosphorylated at thr220/179 of Smad2/3.^15^, ^16^ The application of antibody (Abs) reactive with structurally related phosphorylated peptides reveals that there are three types of phospho‐isoforms: C‐terminally phosphorylated Smad2/3(pSmad2C and pSmad3C), linker‐phosphorylated Smad2/3(pSmad2L and pSmad3L) and dully phosphorylated Smad2/3(pSmad2L/C and pSmad3L/C).^17^ Different phosphorylated forms of Smad2/3 guide different gene expression and show different biological effects. TβRI phosphorylates COOH‐tail serine residues of Smad2 and Smad3. Both pSmad2C and pSmad3C translocate with Smad4 to the nucleus. Smad2/3/4 complex binds the p21^waf1^ promoter and suppresses cell growth. On the other hand, pro‐inflammatory cytokines (CKs), such as tumor necrosis factor‐α (TNF‐α) activate c‐Jun N‐terminal kinase (JNK), which phosphorylated the linker regions of Smad2 and Smad3. pSmad3L translocate with Smad4 to nucleus and binds plasminogen activator inhibitor type 1(PAI‐1) promoter. PSmad2L is localized in the cytoplasm, and Smad2 translocates to the nucleus only after COOH‐tail phosphorylated by TβRI. PSmad2L/C in cooperation with pSmad3L and Smad4 stimulated PAI‐1 transcription and ECM deposition. In addition, pSmad3L upregulates c‐Myc and stimulates cell growth.^14^

## 2 HIPPO‐YAP/TAZ SIGNALING PATHWAY

### 2.1 The function of the Hippo‐YAP/TAZ signaling pathway

The Hippo‐YAP/TAZ signaling pathway was first identified in *Drosophila melanogaster*, and its main role is to regulate the proliferation, differentiation, and migration of cells in organs. Dysregulation of the Hippo‐YAP/TAZ signaling pathway leads to abnormal cell growth and tumor formation. Sakabe et al. showed that YAP and TAZ were necessary for the proliferation of endothelial cells during retinal angiogenesis. In addition, YAP in the cytoplasm promotes endothelial cell migration.^18^

### 2.2 Regulation mechanism of the Hippo‐YAP/TAZ signaling pathway

**Figure 3:**
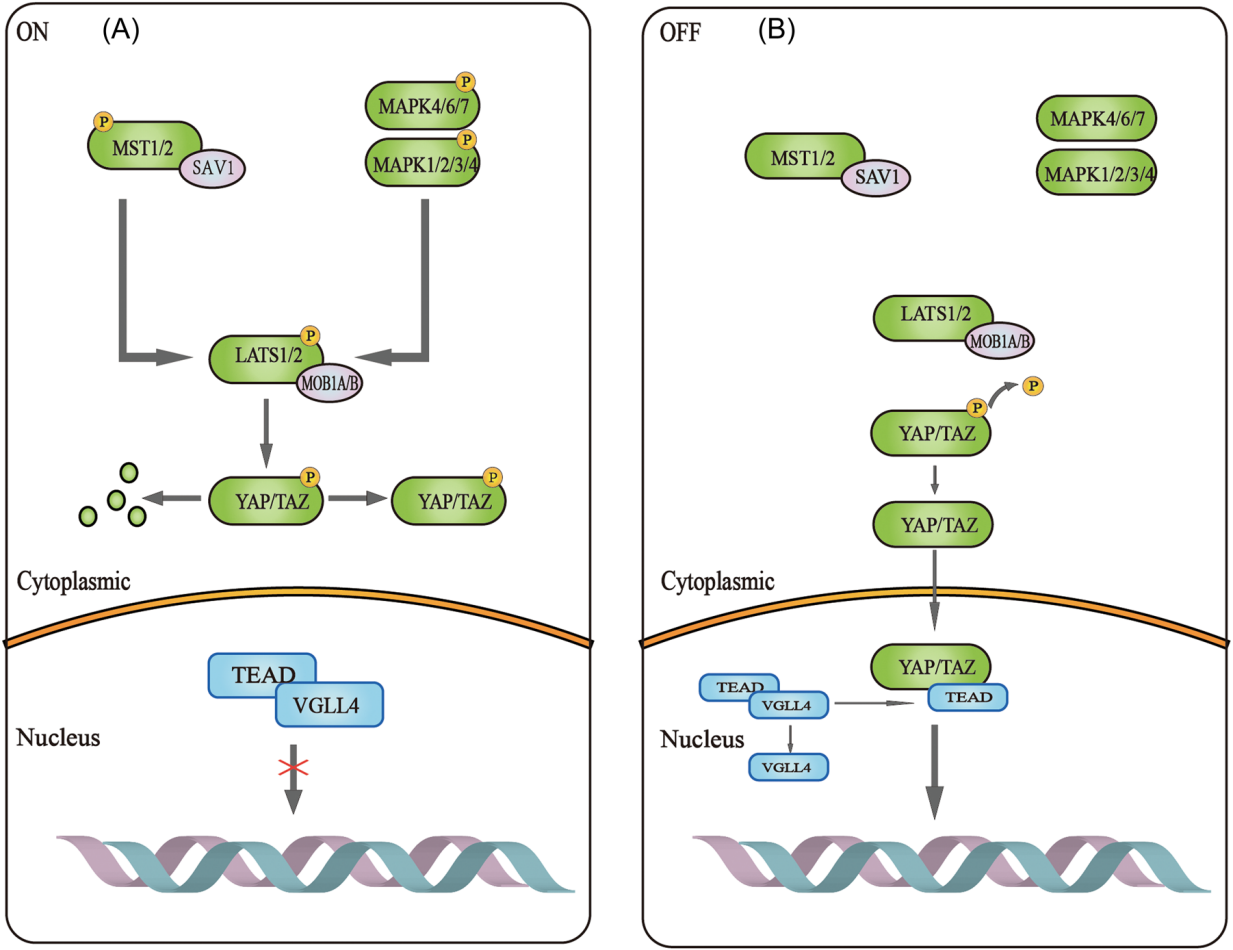
Mechanism of Hippo signaling pathway. (A) When the Hippo pathway is activated, LATS are phosphorylated, which phosphorylates and prevents YAP/TAZ translocation into the nucleus, resulting in YAP/TAZ remaining in the cytoplasm or being degraded. At this time, TEAD in the nucleus binds to VGLL4 and acts as a transcriptional silencer. (B) When Hippo signaling is turned off, phosphorylated YAP/TAZ is dephosphorylated and incorporated into the nucleus. At this time, TEAD in the nucleus is separated from VGLL4 and forms a complex with YAP/TAZ to mediate gene transcription and expression.

When the Hippo‐YAP/TAZ signaling pathway is activated by TAO kinase (Figure 3A), MST1/2 in the pathway is phosphorylated to form LATS1/2 by the costimulation of the scaffold proteins SAV1, MOB1A/B and NF2, while MAP4K4/6/7 and MAP4K1/2/3/5 in the pathway are activated and phosphorylated by NF2 to form LATS1/2. Phosphorylated LATS1/2 activates and phosphorylates YAP/TAZ to cause them to stay in the cytoplasm or be degraded by SCF.^5^ The Hippo‐YAP/TAZ signaling pathway is activated to limit growth and cell proliferation. When the Hippo‐YAP/TAZ signaling pathway is in an inactivated state (Figure 3B), YAP and TAZ are dephosphorylated and translocated into the nucleus. In the absence of nuclear YAP/TAZ, TEAD binds to VGLL4 and acts as a transcriptional silencer. When YAP and TAZ are translocated into the nucleus, TEAD1–4 are separated from VGLL4, and then YAP and TAZ bind to TEAD1–4 instead of VGLL4. TEAD‐mediated gene transcription is activated, which can promote tissue growth and inhibit apoptosis.^19^

## 3 VASCULAR ENDOTHELIAL GROWTH FACTOR

### 3.1 Vascular endothelial growth factor function

Vascular endothelial growth factor (VEGF) is a homodimeric glycoprotein with a molecular weight of 45 kDa.^20^ VEGF mainly plays a role in promoting angiogenesis in the embryonic stage, while it mainly plays a role in repairing pathological vascular injury in the adult stage.^21^ It plays an important role in angiogenesis, the repair of vascular injury, and the maintenance of vascular health, and the mechanisms include inhibition of the proliferation of vascular smooth muscle cells (VSMCs), improvements in endothelial cell survival rate, and the inhibition of thrombosis and inflammation in the vascular wall.^22^

### 3.2 Mechanism of VEGF action

The VEGF family consists of multiple members: VEGF‐A (with multiple subtypes), VEGF‐B, VEGF‐C, VEGF‐D, VEGF‐E, VEGF‐F, placental growth factor, and the newly discovered endocrine‐derived vascular endothelial growth factor (EG‐VEGF).^23^, ^24^ VEGF receptors have three types: VEGFR‐1, VEGFR‐2 and VEGFR‐3. VEGFR‐1 and VEGFR‐2 are mainly expressed in vascular endothelial cells, but they can also be expressed in nonendothelial cells. However, VEGFR‐3 is mostly expressed in endothelial lymphocytes. VEGF gene expression is regulated by a variety of growth factors, such as fibroblast growth factor (FGF), epidermal growth factor (EGF), and tumor necrosis factor (TNF). In addition, hypoxia is another inducing factor.^25^ VEGFs trigger signal transduction by binding to receptors and promoting receptor phosphorylation. Compared with VEGFR‐1, VEGFR‐2 has stronger angiogenesis‐promoting activity and higher tyrosine kinase activity.^26^

## 4 INTERACTIONS OF THE TGF‐β/SMADs SIGNALING PATHWAY, HIPPO‐YAP/TAZ SIGNALING PATHWAY, AND VEGF

### 4.1 Interactions between the TGF‐β/Smads signaling pathway and HIPPO‐YAP/TAZ signaling pathway

**Figure 4:**
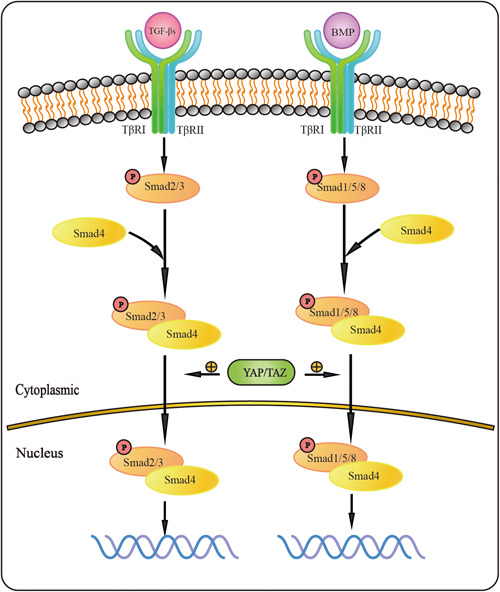
Interaction between TGF‐β signaling pathway and Hippo signaling pathway. There is an interaction between Hippo signaling pathway and TGF signaling pathway, which together play a role in vascular remodeling. The main mechanism is that YAP/TAZ promotes TGF‐β signaling pathway by promoting the translocation of R‐Smad and Co‐Smad complex into the nucleus.

YAP/TAZ plays a role in vascular development, and studies have shown that TAZ promotes SMC differentiation through a nonclassical TGF‐β/Smads signaling pathway.^27^ The interaction of YAP and Smad2 is stimulated by TGF‐β and is necessary for nuclear translocation (Figure 4). The binding of YAP/TAZ to Smad2 is essential for Smad2 nuclear translocation and efficient TGF‐β transcription.^28^, ^29^, ^30^ Studies have shown that YAP, Smad2/3, and TEAD work together to regulate TGF‐β‐induced transcriptional programs to induce the migration and invasion of breast cancer.^31^ It has also been found that the YAP/TEAD/Smad3/p300 complexes can drive the upregulation of the Ctgf gene, thereby regulating cell proliferation and ECM production.^32^ The interaction between YAP1 and Smad2 can promote nuclear translocation of Smad2 and activation of the TGF‐β/Smads pathway, leading to intracellular invasion.^33^ However, YAP and TAZ were proven to bind to Smad2/3 in the TGF‐β/Smads signaling pathway and form complexes that affect Smad nuclear/mass conversion by isolating Smads in the cytoplasm.^34^ Studies have shown that BMP2, a member of the TGF‐β superfamily, can promote the phosphorylation of downstream Smad 1/5/8, thus increasing the protein level of SMAD1. Nevertheless, when the expression level of YAP is decreased, the phosphorylation of Smad1/5/8 and the protein level of Smad1 are decreased.^35^ Existing studies have shown that the Hippo‐YAP/TAZ signaling pathway may regulate the TGF‐β/Smads signaling pathway by regulating Smad nucleation and TGF‐β/Smads signaling pathway activity.^36^ In summary, the downstream signaling molecules YAP and TAZ of the Hippo‐YAP/TAZ signaling pathway promote the transduction of the TGF‐β/Smads signaling pathway by regulating the translocation of Smads, the downstream signaling molecules of the TGF‐β/Smads signaling pathway, into the nucleus, which establishes the interaction between the two pathways.

### 4.2 Interaction between the Hippo‐YAP/TAZ signaling pathway and VEGF

**Figure 5:**
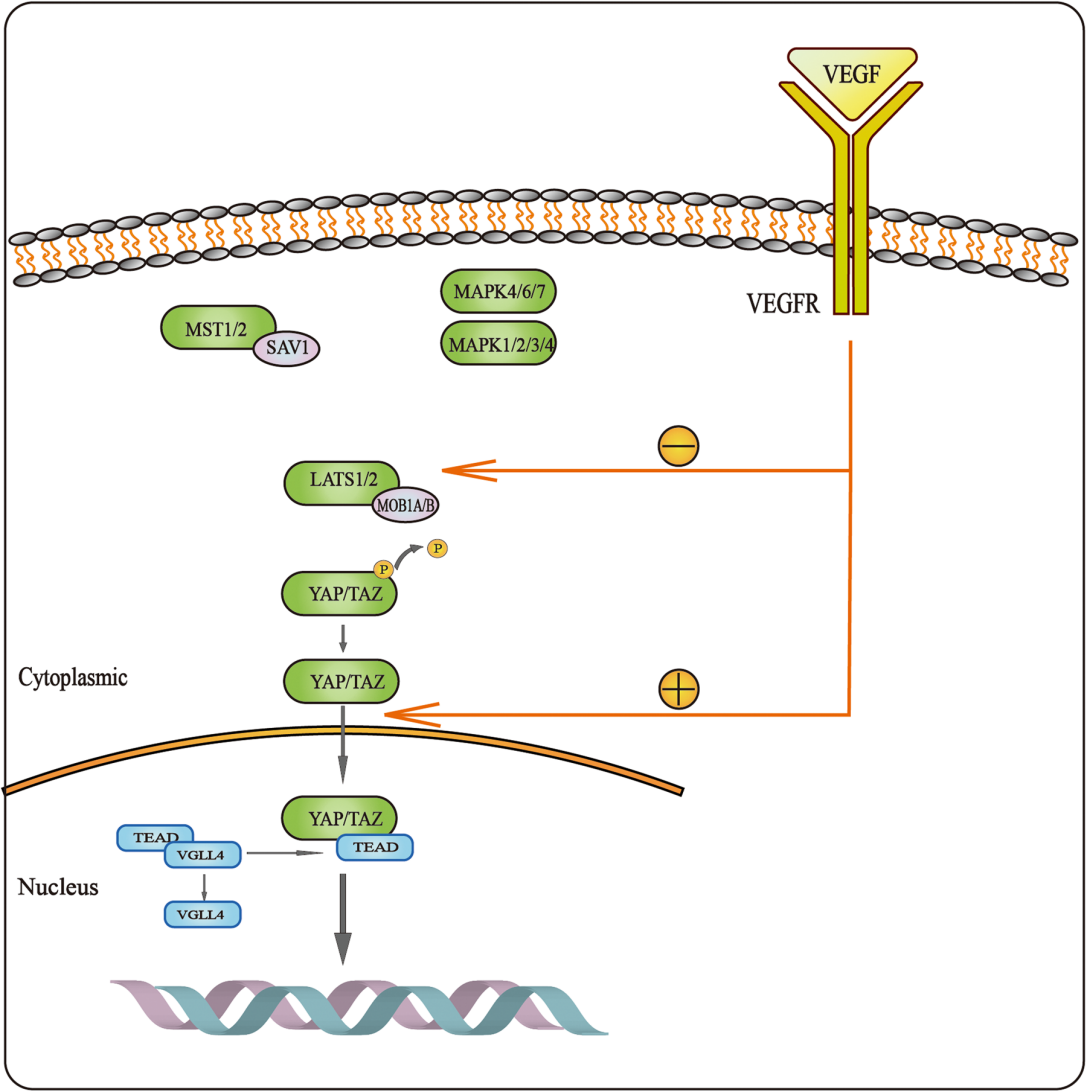
Interaction between Hippo signaling pathway and VEGF/VEGFR. VEGF stimulation leads to cytoskeletal changes and promotes YAP/TAZ activation and nuclear entry, which in turn regulates the expression of related proteins and affects cytoskeletal movement. VEGF/VEGFR promotes YAP/TAZ translocation to the nucleus by inhibiting the phosphorylation of LATS.

The Hippo‐YAP/TAZ signaling pathway plays an important role in VEGF‐induced angiogenesis and development. VEGF stimulation leads to cytoskeletal changes and promotes YAP/TAZ activation and nuclear entry, which in turn regulates the expression of related proteins and affects cytoskeletal movement (Figure 5). Studies have shown that endothelial cell knockout of YAP/TAZ can reduce the expression of the proteins MACF1 and MYO1C, which are involved in cytoskeletal reconstruction and protein transport and block the transport of VEGFR2 protein, thus inhibiting angiogenesis.^37^, ^38^ The Hippo‐YAP/TAZ signaling pathway is a key regulator of VEGF/VEGFR‐induced angiogenesis and vasculogenic mimicry (VM). Azad et al.^39^ found that various VEGFR inhibitors could activate LATS in the Hippo‐YAP/TAZ signaling pathway and inhibit YAP/TAZ. Among them, SU4312 had the most significant inhibitory effect. The mRNA levels of CYR61 and CTGF, two downstream transcriptional targets of YAP/TAZ, were also significantly reduced after VEGFR inhibitor treatment. Three types of cells with high expression of YAP or TAZ were treated with VEGF, and the subcellular localization of YAP/TAZ was determined. Nuclear translocation of YAP/TAZ occurred within 13–30 min after VEGF treatment. Since LATS inhibits YAP/TAZ by phosphorylating and chelating YAP/TAZ in the cytoplasm, this finding suggests that VEGF/VEGFR can activate YAP/TAZ and promote YAP/TAZ nuclear translocation by inhibiting LATS. Further experiments showed that the inhibitory effect may be achieved by P13K/MAPK‐mediated inhibition of MST1/2. Studies^37^, ^39^ have shown that VEGF‐treated endothelial cells can promote the translocation of YAP into the nucleus, while knocking down YAP can reduce the proliferation, migration, and angiogenesis of VEGF‐induced endothelial cells.

## 5 VASCULAR REMODELING

Vascular remodeling is a change in vessel wall structure, which often occurs under pressure, inflammation, trauma, and other stimulation conditions. Oxidative stress and inflammation are often the pivotal players in endothelial dysfunction during abnormal vascular remodeling.^40^ Vascular remodeling is a process driven by all types of vascular cells and participates in the reconstruction of the extracellular matrix (ECM). Positive and negative remodeling is based on how those stimulus factors coordinate and determine the direction of remodeling.^41^ VSMCs are the main structural cells of the vascular wall. Under pathological conditions such as hypertension, the proliferation, migration, and phenotypic changes of VSMCs lead to vascular remodeling. In addition, the accumulation of inflammatory cells, especially monocytes and macrophages, plays a key role in vascular remodeling by regulating SMC function and ECM turnover^42^ (Figure 5).

### 5.1 Relationship between the TGF‐β/Smads signaling pathway and vascular remodeling

**Figure 6:**
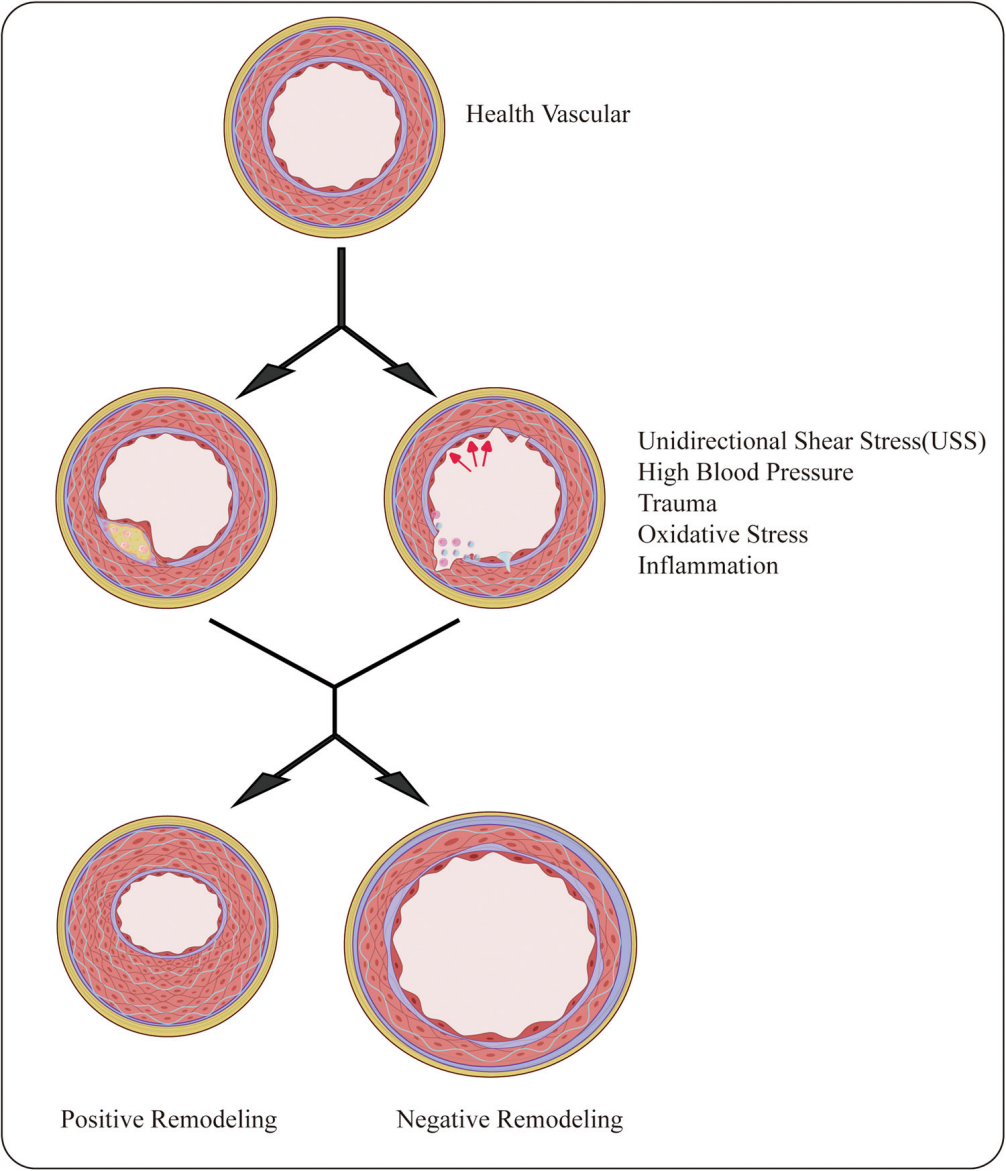
Vascular remodeling process. Changes in vascular anatomy, vascular microenvironment, and hemodynamics are brought on by stimulation from various sources, including hypertension, trauma, oxidative stress response, inflammation, etc. Inflammation and oxidative stress are thought to play a more important role in driving vascular remodeling. To adapt to the changes, vascular remodeling is driven. However, excessive vascular remodeling is the key factor speeding up the development of disorders associated with vascular remodeling. Vascular remodeling includes positive and negative remodeling which is determined by the interaction of different stimuli.

TGF‐β/Smads signaling pathway has different biological effects on angiogenesis in different pathological environments and concentrations and can inhibit or promote angiogenesis.^43^ TGF‐β1 has been shown to promote angiogenesis by upregulating thrombopoietin‐4 (TSP‐4) through a Smad3‐mediated signaling pathway.^44^ Angiotensin receptor‐like kinase 1 (ALK1) is an endothelial‐specific TGF‐β type I receptor that activates the downstream Smad1/5/8 signaling factor, which binds to Smad4 to form a complex, enters the nucleus, and regulates gene expression. This factor may increase the recruitment of pericytes by increasing PDGF‐BB expression in endothelial cells, thereby inhibiting the degradation of the ECM and promoting angiogenesis.^36^ Congenital diaphragmatic hernia (CDH) is a pulmonary dysplasia characterized by reduced distal airway branches, reduced alveolar numbers, and thickening of the lung wall. Ullrich et al.^45^ found that the expression of microRNA(miR)200b in tracheal secretions of fetuses that survived fetoscopic endoluminal tracheal occlusion treatment was higher than that of fetuses that did not respond to tracheal occlusion. The microRNA200 family inhibits several genes in the TGF‐β pathway. Through in vitro studies, they found that treatment of miR200b can induce TGF‐β signaling pathways and increase the occurrence of branching morphologies. In their study, they delivered miR200b in utero using PACE NPs to treat a rat model of CDH, and they found that in utero delivery of miR200b induces alterations in the TGF‐β pathway, leads to pulmonary vascular remodeling, and improves PH.^45^ Similarly, a recent experiment by Da et al. on the treatment of abdominal aortic aneurysms (TAA) in mice found that the application of AGGF could inhibit vascular inflammation and remodeling of TAA by blocking the cleavage of LAP‐TGF‐β1 to form mature TGF‐β1 and inhibiting Smad2/3 and ERK1/2 phosphorylation in VSMCs.^46^ Their experiments both elucidate the crucial role of TGF‐β signaling pathway in angiogenesis and vascular remodeling (Figure 6).

### 5.2 Participation of the Hippo‐YAP/TAZ signaling pathway in vascular remodeling

YAP/TAZ in endothelial cells is an important regulator of angiogenesis in the growth and development of the embryo and postnatal life. This important role of YAP/TAZ was first expressed in the central nervous system after birth. As the body grows and develops, this effect will also be observed in the liver and other organs.^37^ Studies have shown that mice with specific knockout of YAP/TAZ have a decrease in the number of apical cells and vascular sprouting. YAP/TAZ is involved in the formation of new blood vessels in the embryonic stage. However, its role of angiogenesis in adulthood is not obvious, but it plays an important role in pathological vascular remodeling.^38^ Liu H.T. et al. found that knockout of HSF110 improved hypoxia‐induced pulmonary artery remodeling in PH mice by inhibiting the YAP/TAZ‐TEAD4 pathway, thereby improving PH disease progression.^47^ Ong et al. found that ECs lacking YAP/TAZ or their transcriptional partners, TEAD1, 2, and 4 fail to divide, resulting in stunted vascular growth in mice. They also found that YAP/TAZ promote angiogenesis by fueling nutrient‐dependent mTORC1 signaling.^48^ Studies have shown that endothelial cell YAP/TAZ can inhibit inflammation and delay the formation of atherosclerosis. YAP/TAZ inhibits the JNK signaling pathway and downregulates pro‐inflammatory gene expression, thereby reducing monocyte attachment and infiltration.^49^

### 5.3 VEGF involvement in vascular remodeling process

Under various stimulation conditions, VEGF, angiotensin II (Ang II), and FGF induce the activation of matrix proteolytic enzymes (ECM), resulting in the degradation of ECM and the shedding of pericytes on the vascular wall. VEGF contributes to the remodeling of the ECM by increasing the permeability of the vascular endothelium, leading to the extravasation of plasma proteins and deposition in the ECM.^50^ VEGFR‐2 regulates endothelial cell migration, proliferation, differentiation, and survival, as well as vascular permeability and dilation.^51^ Studies have shown that VEGF can promote the repair of damaged blood vessels by indirectly inducing SMC migration by upregulating FGF‐2 levels and downregulating TGF‐β1 levels. FGF‐2 plays a vital role in cell proliferation, differentiation, migration, angiogenesis, and wound healing.^22^ Mice with VEGF allele knockout die because of vascular developmental disorders of the embryo and yolk sac.^52^ Burgers et al. found that vioA profoundly reduced the vascular leakage and EC proliferation after laser‐induced choroidal neovascularization in vivo and the microvascular sprouting of choroidal explant cultures ex vivo. The effects of vioA were mechanistically based on an impairment of the VEGFR2 signaling pathway.^53^ VEGF plays an important role in angiogenesis and pathological vascular repair and remodeling.

## 6 VASCULAR REMODELING‐RELATED DISEASES

At present, the occurrence and development of many diseases are related to vascular remodeling, such as atherosclerosis, hypertension, aneurysm and arterial dissection, and vertebrobasilar dolichoectasia (VBD).

### 6.1 TGF‐β/Smads signaling pathway, Hippo‐YAP/TAZ signaling pathway, and VEGF, as well as their roles in vascular remodeling of atherosclerosis

Vascular remodeling can be classified as positive or negative remodeling according to the remodeling index (RI). The RI is the ratio of the cross‐sectional area (CSA) of the vascular external elastic membrane (EEM) at the lesion to the CSA of the EEM of the proximal or distal vessel. RI > 1.05 indicates positive remodeling, RI < 0.95 indicates negative remodeling, and 0.95 > RI > 1.05 indicates intermediate remodeling. Initially, it was showed that positive vascular remodeling was more beneficial to atherosclerosis than negative vascular remodeling. However, with further research, it has been found that although positive vascular remodeling can compensate for luminal stenosis, the plaques of patients with positive vascular remodeling are more prone to thrombosis than those with negative vascular remodeling.^54^ Positively remodeled vascular plaques contain more macrophages, larger necrotic cores, and thin fibrous caps, which are prone to breakage. Plaques with negative remodeling are mainly fibrocalcified, and the plaques are more stable.^54^, ^55^ The damage of positive remodeling in vascular plaques may be related to the cytokines secreted by a large number of inflammatory cells in the plaque, such as hypoxia‐inducible factor, VEGF, and matrix metalloproteinases (MMPs). These cytokines can promote neovascularization in blood vessels. Recent studies reveal an essential role of the Hippo‐YAP/TAZ pathway in atherogenesis, angiogenesis, and vascular remodeling. YAP and TAZ are regulated by shear stress in endothelial cells.^49^, ^56^

### 6.2 TGF‐β/Smads signaling pathway, Hippo‐YAP/TAZ signaling pathway, and VEGF, as well as their roles in vascular remodeling of hypertension

In hypertension and hypertensive diseases, arterial remodeling is stimulated by hemodynamic changes, resulting in reduced vascular compliance. The remodeling process is mainly changed by endothelial dysfunction, the proliferation and migration of medial VSMCs, the apoptosis and transformation of extravascular fibroblasts, and the synthesis, degradation, and rearrangement of the ECM.

Endothelium‐dependent vasodilation is impaired in animal models of balloon injury‐induced vascular remodeling, so vascular remodeling is thought to lead to endothelial dysfunction.^57^ During the progression of hypertension, the proliferation and migration of VSMCs increase, leading to the loss of contractility and abnormal ECM.^58^ In the process of vascular remodeling, adventitial fibroblasts (AFs) are activated and differentiate into myofibroblasts (MFs), which proliferate and migrate to the media and intima, participating in the process of vascular remodeling. This process leads to fibroblast proliferation and tissue fibrosis through a non‐Smad‐dependent TGF‐β pathway.^59^ ECM synthesis and degradation are dynamic processes that exist throughout vascular remodeling. As a basic structural element of the ECM, collagen limits the overexpansion of the vessel. Collagen I, III, and IV deposition in the blood vessel wall was increased in a mouse model of hypertension.^60^, ^61^ Evidence suggests that several prohypertensive factors and signaling pathways are involved in collagen overproduction. These include TGF‐β‐stimulated Smad2/3 and mitogen‐activated protein kinase (MAPK) pathways, Ang II‐activated activator protein‐1 (AP‐1), protein kinase C (PKC), and p38 MAPK signaling.^62^, ^63^ Jia et al. used Liensinine to intervene in Ang II‐induced hypertension mice and found that Liensinine could improve the vascular remodeling of mice's abdominal aorta by inhibiting MAPK/TGF‐β1/Smad2/3 signaling pathway. The experiment demonstrated the essential role of TGF‐β/Smad2/3 signaling pathway in vascular remodeling and revealed that inhibition of vascular remodeling may become a new therapeutic strategy to inhibit the progression of hypertensive diseases.^64^

### 6.3 TGF‐β/Smads signaling pathway, Hippo‐YAP/TAZ signaling pathway, and VEGF, as well as their roles in vascular remodeling of aneurysm

Aortic aneurysm formation is mainly due to the weakening of the aortic wall, resulting in aortic enlargement.^65^ The cellular and extracellular components of the aortic wall dynamically change at different stages of aortic pressure, injury repair, and remodeling, thus providing appropriate compliance and sufficient strength to adapt to changes in hemodynamics.^66^ SMCs, which are the main regulatory cells in the vascular wall, undergo phenotypic regulation to control the structure and function of the aorta under conditions of inflammation and injury. The most common phenotypic modulation in aortic aneurysm patients is a decrease in the expression of SMC proteins and an increase in the expression of inflammatory proteins. Phenotypic conversion of SMCs can be regulated by various mechanisms, including transcriptional control, signaling pathway regulation, microRNA regulation, and epigenetic regulation. The TGF‐β/Smads signaling pathway plays an important regulatory role in maintaining aortic contractile phenotype and function. A recent study showed that postnatal mice with disruptions in the gene encoding TβRII showed insufficient contractility and elasticity in their ascending aorta and proximal aorta.^67^ In addition, Smad3 deficiency causes SMCs to switch from a contractile to a synthetic phenotype.^68^

### 6.4 TGF‐β/Smads signaling pathway, Hippo‐YAP/TAZ signaling pathway, and VEGF, as well as their roles in vascular remodeling of VBD

VBD is a rare cerebrovascular disease, and the main manifestations are vertebrobasilar artery prolongation, tortuosity, and dilation.^69^ The pathophysiological mechanism may involve an imbalance in MMP expression leading to the degradation of various matrix proteins in the vascular wall, causing abnormal remodeling of the vascular wall and abnormal proliferation of connective tissue.^70^ Evidence suggests that MMPs can break down the inner elastic membrane and allow SMCs to migrate to the middle layer. The repeated destruction and repair of the inner elastic membrane lead to weakness of the vessel wall, resulting in uneven thickness of the vessel wall and then leading to hemodynamic changes. The vicious cycle caused by pathological vascular remodeling and hemodynamic changes is the main pathophysiological mechanism of VBD. Zhu et al. successfully induced the VBD model and found that the inflammatory cascade also plays an important role in VBD.^71^ The proliferation and migration of vascular parietal cells and the reconstruction of the vascular microenvironment in the process of vascular remodeling may be important reasons for the aggravation of tortuous vascular dilation. The clinical manifestations of VBD are not specific, often manifesting as ischemic stroke, cerebral hemorrhage, hydrocephalus, and compression of the brain stem and cranial nerves, and there are some asymptomatic patients. VBD can cause changes in posterior circulation hemodynamics and abnormal blood perfusion, leading to cerebral ischemia and infarction. At present, there is no effective treatment. It is very important to detect the combined symptoms early and apply early clinical interventions. Although the pathogenesis of VBD is still unclear, it may have the same or a similar mechanism as other vascular remodeling‐related diseases. The TGF‐β/Smads signaling pathway, Hippo‐YAP/TAZ signaling pathway, VEGF, and other angiogenesis‐related factors may play a key role in the process of vascular remodeling leading to vascular extension and expansion.

### 6.5 TGF‐β/Smads signaling pathway, Hippo‐YAP/TAZ signaling pathway, and VEGF, as well as their roles in vascular remodeling of pulmonary hypertension

According to the pathological and hemodynamic characteristics, PH can be divided into five categories, including arterial pulmonary arterial hypertension (PAH), pulmonary hypertension (PH) caused by left heart disease, PH caused by respiratory diseases and hypoxia, PH caused by pulmonary obstructive diseases, PH caused by unknown causes and multifactorial PH. PH caused by respiratory diseases includes interstitial pulmonary diseases, chronic obstructive pulmonary disease (COPD), and sleep apnea. Pulmonary vascular remodeling refers to vascular structural changes caused by injury factors such as hypoxia, high blood shear stress, and inflammation, which are the main pathological features of pulmonary arterial hypertension (PAH). Vascular remodeling in PH is characterized by the accumulation of different vascular cells in the pulmonary artery and the infiltration of inflammatory cells around the vessel.^72^ The basic pathological process of remodeling involves cellular hypertrophy, hyperplasia, inflammation, apoptosis, migration, and accumulation of ECM. These pathological changes result in structural changes in the intima, media, and adventitia of the vessel wall.^73^ VEGF is associated with the whole process of COPD development, is closely related to pulmonary vascular remodeling, and participates in the development of COPD PH. Serum VEGF concentrations can be used as sensitive indicators of the severity, activity, and prognosis of COPD.^74^ SMCs can release a variety of stimulatory cytokines, including TGF‐β1. This factor can actively induce endothelial progenitor cells to migrate to the inner membrane, thereby promoting pulmonary vascular remodeling.^75^ Gore et al. studied the expression of TGF‐β in lung endothelial cells of 14 PAH patients and 15 normal controls and found that the expression level of TGF‐β in lung endothelial cells of PHA patients was significantly increased. The experimental results showed that the TGF‐β signaling pathway directly acted on lung ECs to promote an increase in growth factors and inflammatory factors, which played an important role in hypoxic PH.^76^ Tang et al. used Ginsenoside Rg1 to treat hypoxia‐induced PAH rats and found that hypoxia could promote the inflammation and vascular remodeling of pulmonary artery tissue in rats, and the levels of TGF‐β1 and p‐Smad2/3 in pulmonary artery tissue were significantly upregulated. Vascular remodeling was significantly improved in rats treated with Ginsenoside Rg1, and TGF‐β1 and p‐Smad2/3 levels in pulmonary artery tissue were also reduced. This suggests that inflammation and vascular remodeling play a crucial role in the occurrence and development of PAH diseases and the role of TGF‐β/Smad2/3 signaling pathway in vascular remodeling. Inhibition of TGF signaling can improve vascular remodeling and inhibit the progression of PAH disease.^77^ In addition, ECM remodeling during vascular remodeling can lead to ECM hardening. ECM sclerosis can activate YAP/TAZ to maintain the proliferation and migration of pulmonary artery endothelial cells and pulmonary VSMCs. The mechanism may be that mechanical stimulation leads to YAP/TAZ activation to manipulate metabolic enzymes, including glutaminase, to induce glutamine breakdown and glycolysis.^78^

Although current advances in the treatment of PAH have improved patient outcomes, 5‐year and 10‐year survival rates of 52%–75% and 45%–66%, respectively.^79^ However, these patients still have limited lung function and reduced long‐term survival, which may be due to the role of irreversible vascular remodeling. The signature feature of PAH vascular remodeling is the excessive proliferation and antiapoptosis of pulmonary artery smooth muscle cells. Therefore, inhibiting the over‐remodeling process of PAH vessels may be a new therapeutic strategy to delay the progression of PAH disease and increase the long‐term survival rate of patients.

### 6.6 TGF‐β/Smads signaling pathway, Hippo‐YAP/TAZ signaling pathway, and VEGF, as well as their roles in vascular remodeling of restenosis after percutaneous transluminal angioplasty

Restenosis after percutaneous transluminal angioplasty (PTA) is a major factor affecting the long‐term outcome of this technique. It was previously thought that the occurrence of restenosis after PTA was similar to wound healing. That is, after PTA balloon expansion, vascular wall injury causes vascular elastic retraction, thrombosis, and the migration and proliferation of VSMCs.^80^ Therefore, the endointimal hyperplasia theory was used to explain the stenosis of the lumen during restenosis. However, in recent years, the failure of many clinical trials on intimal hyperplasia has led to a new understanding of the mechanism of restenosis.^81^ At present, current research tends to support that restenosis after PTA is mostly caused by intimal hyperplasia and vascular remodeling rather than only intimal hyperplasia. The results of restenotic vascular remodeling after PTA include compensatory vessel thickening caused by the increase in the whole vessel CSA and chronic retraction caused by the decrease in the whole vessel area. That is, dilated vascular remodeling and contractile vascular remodeling work together.^82^ Similarly, various pathways and factors play important roles in the process of vascular remodeling. Ryan et al. established a balloon rat model of common carotid artery injury and showed that by blocking the effect of TGF‐β with a selective inhibitor of TβR II, the expression of TGF‐β in vascular advection was reduced, and the secretion of type I collagen and SMA in the ECM was significantly reduced. After 2 weeks, the proliferative area of the intima decreased, and the diameter of the lumen increased. This finding suggests that TGF‐β stimulates fibroblast phenotype conversion to SMA‐expressing MFs and stimulates ECM secretion.^83^

## 7 CONCLUSION AND FUTURE PROSPECTS

Although exploration of the pathological mechanism of vascular remodeling has made progress in recent decades, the specific and comprehensive mechanism is still not fully elucidated. This article reviews the possible mechanism of vascular remodeling and its role in the occurrence and development of many diseases according to current research progress. In conclusion, vascular remodeling can be triggered by various stimuli, including high blood pressure, trauma, oxidative stress, and inflammation, to make up for the aberrant changes in hemodynamics and vascular microenvironment brought on by vascular injury. Proper vascular remodeling can compensate for vascular dysfunction and even restore vascular function. On the contrary, excessive vascular remodeling can aggravate the disease progression of vascular dysfunction and vascular remodeling‐related diseases. Treatments for these disorders that reduce inflammation are currently making significant progress. Despite this, there are currently no effective treatments for vascular remodeling. More research is required to determine how to prevent excessive vascular remodeling and slow the progression of disease. Numerous signaling pathways are active during vascular remodeling, according to recent studies. By inhibiting the excessive conduction of these signaling pathways, the occurrence of aberrant vascular remodeling can be prevented and the disease progression of vascular remodeling‐related diseases can be delayed.

The Hippo‐YAP/TAZ signaling pathway, TGF‐β/Smads signaling pathway, and VEGF play important roles in the process of angiogenesis and the repair of pathological damaged blood vessels, and there are certain interactions among them. According to recent research, inhibiting the Hippo‐YAP/TAZ signaling pathway, TGF‐β/Smads signaling pathway, and VEGF can prevent aberrant vascular remodeling and hence slow the progression of illness. On the other hand, we discovered that there is a relationship between the TGF‐β/Smads signaling pathway, the Hippo‐YAP/TAZ signaling pathway, and VEGF. Therefore, inhibiting one of the three may also indirectly inhibit the effect of the other. By studying the mechanism of the three factors in vascular remodeling and related diseases, we hope to provide new therapeutic ideas for vascular remodeling‐related diseases.

## AUTHOR CONTRIBUTIONS

**Hui Liu**: Writing—original draft; writing—review and editing. **Mingyue Sun**: Writing—review and editing. **Nan Wu**: Writing—review and editing. **Bin Liu**: Writing—original draft; writing—review and editing. **Qingxin Liu**: Writing—original draft; writing—review and editing. **Xueli Fan**: Writing—original draft; writing—review and editing.

## CONFLICT OF INTEREST STATEMENT

The authors declare no conflict of interest.
